# Automated Solid-Phase Protein Modification with Integrated Enzymatic Digest for Reaction Validation: Application of a Compartmented Microfluidic Reactor for Rapid Optimization and Analysis of Protein Biotinylation

**DOI:** 10.3389/fbioe.2017.00072

**Published:** 2017-11-13

**Authors:** Regina Fraas, Juliane Diehm, Matthias Franzreb

**Affiliations:** ^1^Institute of Functional Interfaces, Karlsruhe Institute of Technology, Karlsruhe, Germany

**Keywords:** protein modification, solid-phase reaction, enzymatic digest, microfluidic reactor, biotinylation

## Abstract

Protein modification by covalent coupling of small ligands or markers is an important prerequisite for the use of proteins in many applications. Well-known examples are the use of proteins with fluorescent markers in many *in vivo* experiments or the binding of biotinylated antibodies *via* biotin–streptavidin coupling in the frame of numerous bioassays. Multiple protocols were established for the coupling of the respective molecules, e.g., *via* the C and N-terminus, or *via* cysteines and lysines exposed at the protein surface. Still, in most cases the conditions of these standard protocols are only an initial guess. Optimization of the coupling parameters like reagent concentrations, pH, or temperature may strongly increase coupling yield and the biological activity of the modified protein. In order to facilitate the process of optimizing coupling conditions, a method was developed which uses a compartmented microfluidic reactor for the rapid screening of different coupling conditions. In addition, the system allows for the integration of an enzymatic digest of the modified protein directly after modification. In combination with a subsequent MALDI-TOF analysis of the resulting fragments, this gives a fast and detailed picture not only of the number and extent of the generated modifications but also of their position within the protein sequence. The described process was demonstrated for biotinylation of green fluorescent protein. Different biotin-excesses and different pH-values were tested in order to elucidate the influence on the modification extent and pattern. In addition, the results of solid-phase based modifications within the microfluidic reactor were compared to modification patterns resulting from coupling trials with unbound protein. As expected, modification patterns of immobilized proteins showed clear differences to the ones of dissolved proteins.

## Introduction

The synthetic modification of proteins has many applications in biotechnology. By use of chemiluminescent or fluorescent dyes proteins can be made visible *in vitro* and *in vivo* (Gillespie and Hudspeth, 1991; Ballou et al., 1995; Han et al., 2015; Press et al., 2016). Physicochemical properties (solubility, stability) can be altered (Xiao et al., 2008; Morgenstern et al., 2017), or proteins can be activated for immobilization or cross-linking (Tang and Bruce, 2009; Hutsell et al., 2010). A comprehensive and detailed collection of modification reactions and protocols were given in several books and publications (Wu and Goody, 2010; Hermanson, 2013; Boutureira and Bernardes, 2015).

For protein immobilization, biotin plays an important role. Biotin is a 244.31-Da molecule, which takes part in important metabolic processes in nature (McMahon, 2002). It is known for its unusually strong non-covalent interaction with avidin. The dissociation constant is 10^−15^ M (Guesdon et al., 1979) and is thus higher than the one of antibodies and their antigen (Diamandis and Christopoulos, 1991). Avidin is a 66-kDa glycoprotein from eggwhite, consisting of four subunits with 16.4 kDa each (Green, 1975). Each subunit is capable of binding one biotin.

**Figure 1:**
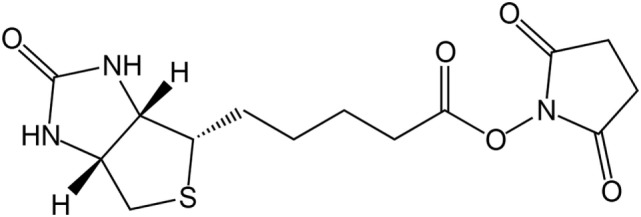
Chemical structure of *N*-hydroxysuccinimide (NHS)–biotin.

Due to the strong bond between biotin and avidin, the biotinylation of proteins is used for affinity chromatography, immunoassays, or immobilization. A well-established reaction for the biotinylation of proteins is the use of reactive groups like the amine-reactive *N*-hydroxysuccinimide (NHS) or the sulfur-reactive maleimide. The structure of biotin–NHS is given in Figure 1. NHS–biotin reacts mainly with the terminal amine or lysines, which are present on the surface of proteins with high frequency (Sokalingam et al., 2013). The reactivity of NHS groups depends on multiple factors like pH-value or temperature which influence the amount of modified lysines. For each protein, screening of these conditions is a necessary prerequisite for yield optimization, but can be time consuming. We developed a compartmented, microfluidic reactor system which allows the parallel evaluation of several reaction conditions for biotinylation. A subsequent on-line enzymatic digest followed by an off-line mass spectrometric measurement allows a fast and detailed analysis of the resulting reaction products.

Compartmentalization of the reactor is achieved in the reaction capillary by inserting aqueous reaction phases followed by non-mixable (organic) solvents or air. For protein modification, the reaction phases consist of aqueous buffers filled with reactants or enzymes. The target protein is immobilized on magnetic particles and can be transported through the reactor. A relative movement between plugs and the immobilized protein is achieved by fixing the magnetic particles with an external bar magnet and pumping the reactor content further until the desired plug is reached. Thus, the particles can sequentially enter reaction plugs filled with different reactants and react accordingly. In Hübner et al. (2015), a precursor of the reactor was presented. In this study, we show an important enhancement of the reactor and its use for the screening of biotinylation conditions. Experiments with free eGFP were conducted under the same conditions in order to show whether differences in the isomer formation occur for the immobilized protein.

## Materials and Methods

### Chemicals and Materials

Chemicals used for this work are given in the following. Sinapic acid, α-cyano-4-hydroxycinnamic acid (CHCA), pepsin from porcine pancreas, trifluoroacetic acid (TFA), biotin NHS ester (NHS-Biotin), and ammonium bicarbonate (NH_4_HCO_3_) were purchased from Sigma Aldrich (St. Louis, MO, USA), Pierce BCA protein assay kit from Thermo Scientific (Waltham, MA, USA).

Albumin fraction V from bovine serum, sodium dihydrogen phosphate dihydrate, ethanolamine, copper(II) sulfate pentahydrate, hydrochloric acid (32%), pH-indicator strips MColorpHast (pH 0–14), pH-indicator strips pH 5–10, and sodium chloride were purchased from Merck Millipore (Darmstadt Germany); dimethyl sulfoxide (DMSO) from VWR (Radnor, PA, USA). Disodium hydrogen phosphate and sodium hydroxide were ordered from Carl Roth GmbH & Co. KG (Karlsruhe, Germany); disuccinimidyl suberate (DSS) was ordered from Cayman Chemical Company (Ann Arbor, MI, USA).

The plasmid for eGFP production was generated at the Institute of Industrial Genetics of the University of Stuttgart, Germany. The plasmid pJOE4056 was used as vector for the fusion of the (His) 6-tag. The protein was expressed by *Escherichia coli* (*E. coli*) BW3110 at the Institute of Biochemical Engineering of the University of Stuttgart, Germany. Fermentation was conducted as described by Wilms et al. (2001).

Magnetic micro particles with iminodiacetic acid functionalization (IDA-particles), with carboxy functionalization (C22) and with *N*,*N*-diethyl-1,3-propanediamine functionalization (DEAP) were kindly donated by PerkinElmer chemagen Technologie GmbH (Baesweiler, Germany). Capillaries consisted of fluorinated ethylene propylene and were purchased from Wolf-Technik (Stuttgart, Germany) with an outer diameter of 3.17 mm and an inner diameter of 1.58 mm.

### Analytical Methods

#### Protein Immobilization and Quantification

The concentration of the bead suspension was determined gravimetrically before protein immobilization. For this, 200 µL of the bead suspension were dried at 60°C in triplicates in pre-weighed HPLC glass vials in a drying oven Heraeus Function Line serie 7000 B6 from Heraeus (Hanau, Germany). After drying of the particles, vials were weighed again on a microbalance Sartorius MC 5 (Göttingen, Germany) and the concentration was calculated. eGFP with His-tag was immobilized on IDA-functionalized particles. The particles were first activated with a solution of 50 mM CuSO_4_, 0.2 M NaCl, pH 4.2 (activation solution). 10 mg particles were mixed with 1.5 mL activation solution at 25°C and 1,400 rpm for 1 h. Particles were washed with 50 mM phosphate buffer pH 7.5 (storage buffer) once and between 0.3 and 1.6 mg protein in solution were added. Immobilization was conducted for 1 h at 25°C and 1,400 rpm. A Thermomixer comfort 5355 by Eppendorf AG (Hamburg, Germany) was used for mixing and tempering of immobilization and modification reactions in reaction vessels.

Afterward, the beads were washed with 10 mM phosphate buffer, 0.8 M NaCl, pH 7.5 (washing buffer) three times, twice with storage buffer and the supernatants were collected. By use of a BCA-Assay protein concentrations in the washing supernatants and the eGFP stock solution were determined. BSA solutions with different concentrations in the same buffer were applied for calibration. Absorption was measured in multititer plates with 96 wells from Brand (Wertheim, Deutschland) in a plate reader EnSpire 2300 Multimode from PerkinElmer (Waltham, MA, USA).

Protein loading was calculated from the difference of the protein mass applied for immobilization (*m*_Protein_) and the protein mass not bound (*m*_Protein, supernatant_) and washed off (*m*_Protein, wash_). The difference was divided by the particle mass (*m*_Particle_), as described in Eq. 1.

(1) $$\text{Load}=\frac{m_{\text{Protein}}-(m_{\text{Protein},\text{Supernatant}}+m_{\text{Protein},\text{Wash}})}{m_{\text{Particle}}}.$$

#### Mass Spectrometric Measurements

Mass spectrometric measurements were performed in a MALDI TOF/TOF 4800 analyzer (Applied Biosystems, Framingham, MA, USA), either in linear mode or in reflector mode. Samples for MALDI-measurements were diluted 1:25 with the matrix solution. For the linear mode, 10 mg/mL sinapic acid in 50% (v/v) acetonitrile with 0.1% TFA were used as matrix, for the reflector mode 10 g/L CHCA in 50% acetonitrile with 1% TFA. 0.5–1 µL of each sample-matrix solution and a calibration mix were spotted onto a MALDI stainless steel target. Dried spots were measured *via* MALDI TOF/TOF. MALDI measurements were analyzed with Data Explorer Software 4.0 (Applied Biosystems). Further analysis was done with the open source software mmass (Strohalm et al., 2010). Spectra in linear mode were smoothed using a Savitzky–Golay filter with a window size of 50 *m*/*z* and five cycles in the software “mmass.”

For measurements in reflector mode, a list of the peptide masses in the spectrum was generated by the built-in tool in the Data Explorer Software 4.0. In order to prove the successful proteolytic digest of the protein, the generated list was entered in the databank “MASCOT Peptide Mass Fingerprint” (Perkins et al., 1999). This software calculates which organism correlates most with the entered data. For the determination of peptide fragments after an enzymatic digest the tool “PeptideMass” from Expasy was used (Wilkins et al., 1997).

### Digest of Free and Immobilized Protein

The analysis of the modified amino acids requires a fragmentation of the protein, which is achieved here with the protease pepsin. For the digest of immobilized protein 0.3 mg eGFP on IDA-particles were applied. The protein was immobilized and then washed with 0.1 M citrate buffer pH 2. The buffer was replaced by 100 µL of a 1 g/L pepsin solution in 0.1 M citrate buffer pH 2. The samples were digested for 21–22 h at room temperature on a shaker. The reaction was stopped by addition of 30 µL of 1 M NaOH per 100 µL sample and prepared for MALDI measurement.

For free protein, buffer exchange was performed using centrifugal concentrators Vivaspin^®^. The buffer of 0.3 mg of free protein was replaced by 0.1 M citrate buffer pH 2 and 100 µL of 1 g/L pepsin solution were added. The following sample preparation corresponded to the one for immobilized protein.

### Determination of Modification Sites

**Figure 2:**
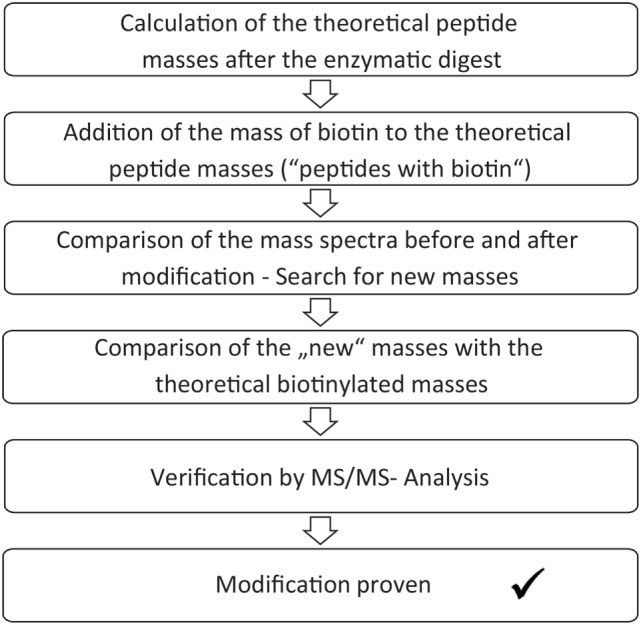
Scheme for analysis of the biotinylated modification sites.

The enzymatic digest and mass spectrometric analysis of the digest product can be used for determination of the biotinylated lysines. Identification of the modification sites occurred according to a protocol, which is summarized in Figure 2. First, the theoretic cleavage sites of pepsin for eGFP were determined using “PeptideMass” from Expasy. The resulting peptide fragments and their masses were calculated with this information. The mass of bound biotin (226.078 g/mol) was added to the theoretic peptide masses and the masses were deposited in a file (“peptides with biotin”). The mass spectra of the unmodified eGFP were compared to the spectra of the modified eGFP afterward in order to find differences. Lists were created with those peptide masses, which were only present in the spectra of the modified proteins (“potentially biotinylated peptides”). The potentially biotinylated peptides were compared to the list of “peptides with biotin.” Agreements were only assumed with mass differences below 0.2 Da and with at least one lysine in the peptide sequence. Additionally, it had to be found in all spectra of the triplicates per run. For verification, MS/MS Measurements were performed. MS/MS measurements allow for a more detailed fragmentation of a mass that was found in the spectrum of a sample. This delivers further information about the peptide. A voltage of 1 kV was used for fragmentation. Only if the MS/MS measurements confirmed the modification of a peptide, a successful reaction was assumed.

### Protein Modification Reactions

Protein modification was either performed using different pH-values or different molar excesses of NHS-biotin compared to the applied eGFP. For the excess-experiments NHS-biotin was used in a 10-, 20-, 50-, or 100-fold molar excess compared to the 0.1 mg immobilized protein per reaction. The particles were inserted into the reactor in a 10-g/L suspension in storage buffer with 15% DMSO. A stock solution with 1.8 g/L Biotin-NHS was made and diluted 1:20 in a solution of 40% DMSO and 60% storage buffer. For reduction of the biotin excess the stock solution was further diluted.

Mixing was achieved by pumping 50 µL with a flow rate of 20 µL/s forth and back. The digest was performed in the reactor as described in Section “Digest of Free and Immobilized Protein.” For comparison, the reaction was also conducted with free protein in micro centrifuge tubes under equal conditions. This reaction was stopped by adding 100 µL of an ethanolamine solution pH 7 with a 30-fold excess compared to biotin.

The experiments for screening of pH-values were performed using 0.3 mg immobilized eGFP. The magnetic particles with the immobilized GFP were pumped into the reactor in a suspension with storage buffer and 15% DMSO. Depending on the desired pH-value, magnetic ion exchange particles were also pumped into the reactor in another plug. They were then moved to the reaction plug, which contained a 20-fold molar excess of biotin-NHS (compared to 0.3 mg eGFP). By mixing the particles in the reaction plug for 5 min, the pH was changed as desired (see Change of pH-Value in the Reactor Device). After removal of the ion exchange particles, the particles with the immobilized eGFP were moved to the reaction plug and the modification took place for 5 min. After the reaction, the particles were pumped out of the reactor and washed. The pH-value of the plug was controlled with pH-indicator strips. Half of the particles were used for protein elution in a solution 0.5% NH_4_HCO_3_ in water twice for 15 min and measured in linear mode. The other half was digested by pepsin and measured *via* MALDI in reflector mode. The reaction was also performed with free protein under the same conditions. This reaction was stopped by adding 100 µL of an ethanolamine solution pH 7 with a 30-fold excess compared to biotin and then prepared for digest and analysis as given in Section “Digest of Free and Immobilized Protein.”

### Change of pH-Value in the Reactor Device

Magnetic ion exchange particles were used to adjust the pH-value in selected plugs inside the reactor. 3 mg particles with carboxy functionalization (C22) could be used to lower the pH-value of 100 µL storage buffer to pH 6.5. 3.8 mg particles with *N,N*-diethyl-1,3-propanediamine functionalization (DEAP) increased the pH-value of 100 µL storage buffer to pH 8. C22 particles were first activated by shaking 54 mg particles in 2 mL 1 M HCl for 10 min. 62 mg DEAP particles were shaken for 10 min in 2 mL 1 M NaOH. The particles were washed with MilliQ and stored therein.

### Titration Curves of Magnetic Particles

The titration curves of the magnetic particles were determined as described by Paulus et al. (2015). For this 20 mg particles were washed in a solution of 0.01 M NaOH and then resuspended in 10 mL of the same solution. A solution of 0.1 M HCl was added step-wise. The particles were separated by use of a magnet and the alteration of the pH-value was measured. Both DEAP and C22 particles were used and titration curves were determined twice. The pKa was determined at the inflection point between the plateaus (Stipanuk and Caudil, 2012). For the DEAP particles, the method of Frimmel et al. (1985) was applied additionally. First, the titration curve for water was substracted from the DEAP titration curve. In order to achieve this, interpolations were performed for the curves, respectively, and the difference of the curves was plotted versus the pH-value. The derivative of the resulting difference plot was formed. For calculating the derivative, the difference plot was approximated to a ninth order polynomic function. The maximums of the derivative plot represent the pKa.

## Results and Discussion

### Reaction Device

**Figure 3:**
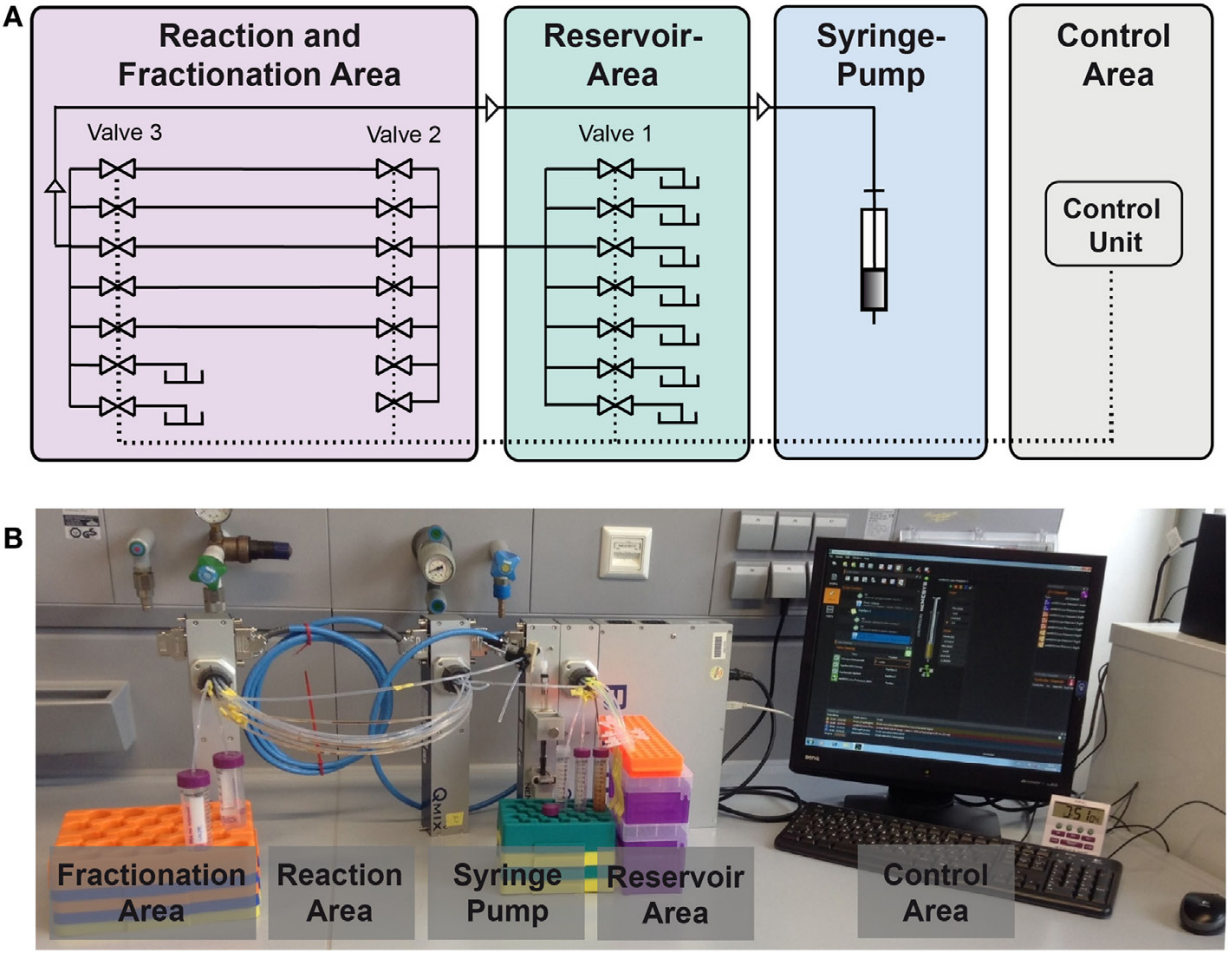
Setup of the compartmented reactor system. **(A)** Scheme showing the different areas. **(B)** Photo showing the actual system.

In this study, we present an enhanced version of the reaction device presented in Hübner et al. (2015). A schematic is given in Figure 3. The syringe pump in “pulling mode” pulls the liquids from the reservoir area (storage of buffers, solvents, and reactant solutions) into the reaction area. In the reaction area, the reaction capillaries are arranged between valve 2 and valve 3 and are filled with reaction and separation plugs. Other than depicted in the scheme, each valve contains 10 ports. As two ports of valve 3 serve as output ports, eight reaction capillaries are available for reactions. The syringe pump and the valves are controlled by the software QmixElements (Cetoni, Korbußen, Germany), thus allowing a semi-automated operation of the reaction device.

The main control unit as well as the modules with a syringe pump or multiport valves, respectively, were purchased from cetoni GmbH (Korbußen, Germany) and consisted of a Qmix base module “BASE 600,” three low pressure valve modules “Qmix V EX” and a “neMESYS” low pressure syringe pump. Whereas in former studies only one reaction capillary was present, parallelization is achieved here by use of three valves. This allows for the introduction of eight reaction capillaries which are usable in parallel. This leads to a significant reduction of experiment duration. In the original version of the device mixing of the magnetic beads in the reactor was mainly achieved by Helmholtz coils inducing a magnetic field. In the case of parallelized reaction capillaries efficient mixing is achieved by pumping the reactor content forth and back for a few microliters. The particles are dispersed inside the plug by this movement. This way, scale-up of the electromagnetic coils and the requirement of cooling of the coils is not necessary anymore.

### Digest and Identification of the Immobilized Protein

eGFP is a very stable protein that is hardly digestible at neutral pH-values (Chiang et al., 2001). A digest can, however, be performed in an acidic medium (Alkaabi et al., 2005), e.g., by the protease pepsin at pH 2. This reaction was already used with free eGFP as a sensor for pepsin activity (Malik et al., 2005). Before the MALDI-TOF analysis could be used for process validation, the applicability of a solid-phase digest by pepsin needed to be verified. The digest of free and immobilized protein was compared, in order to prove that the reaction is similarly efficient. Samples were prepared as described in Section “Digest of Free and Immobilized Protein” and measured *via* MALDI in the reflector mode. By use of “MASCOT Peptide Mass Fingerprint” and adjustment of the search parameters peptide tolerance (altered between 1–2 Da) and missed cleavages (altered between 0 and 2) the samples were assigned to GFP. Due to the high sequence identity of GFP and eGFP of 98.8%, a successful solid-phase digest and assignment can be assumed for both reactions. The sequence of the used eGFP clone is given in the Supplementary Material.

In order to further compare the digest of the free and the immobilized protein, protein sequence coverage was considered. This value is given by the software during analysis. For this value, the peptides identified during the mass spectrometric measurements are aligned with the amino acid sequence of the considered protein. The protein sequence coverage specifies the portion of amino acids that was covered during this alignment. Therefore, it expresses the efficiency of the digest and detection. For the digest of the free and immobilized protein, this value should be as similar as possible. A protein sequence coverage of 0.53 was determined for the immobilized and 0.56 for the free protein with a peptide tolerance of 0.2 Da and one missed cleavage. These values are very close. Thus, the digest appears to be similarly effective in both systems.

### Analysis of Modification Sites

In the preceding section, it was shown that eGFP can be successfully digested in a solid-phase reaction with an efficiency equal to the digest of free protein. Consequently, an enzymatic digest followed by mass spectrometry can be used to analyze and optimize the reaction conditions for solid-phase biotinylation of eGFP. In a first series of experiments different biotin-excesses were examined, followed by a second series in which the reaction pH was varied.

#### Modification with Different Biotin-Excesses

In this experimental series, the biotin excess was varied by applying a molar ratio of 10, 20, 50, and 100 relative to the applied 0.1 mg immobilized eGFP. After the coupling, the reaction products were digested in parallel and the exact positions of the generated modification were determined following the protocol in Section “Protein Modification Reactions.” The list of modified lysines depending on the applied excess is given in Table 1. NHS-biotin attacks mainly amines, e.g., lysines or the N-terminus of the protein. As the His-tag of the eGFP is located at the N-terminus, biotinylation of the N-terminus is not regarded here. From the list it becomes clear that the lysines are modified in a sorted way: If a lysine is modified for a smaller biotin-excess, it is also modified with a higher excess. This indicates that the order of modification is not random. Additionally, the higher the excess of NHS-Biotin, the more lysines are modified, as was expected.

**Table 1:** Modified lysines for the biotinylation with different excesses of biotin–*N*-hydroxysuccinimide.

Different excesses				Different excesses			
Solid-phase reaction				Reaction with free protein			
10×	20×	50×	100×	10×	20×	50×	100×
K 79	K79	K26	K26	K26	K26	K26	K26
K 107	K85	K79	K79	K85	K85	K79	K79
K 113	K107	K107	K85	K107	K101	K85	K85
K 156	K113	K113	K107	K113	K107	K101	K101
K 158	K126	K126	K113	K126	K113	K107	K107
K 162	K131	K131	K126	K140	K126	K113	K113
	K156	K140	K131	K209	K131	K126	K126
	K158	K156	K140	K214	K140	K131	K131
	K162	K158	K156		K166	K140	K140
	K209	K162	K158		K214	K156	K156
	K214	K209	K162			K158	K158
		K214	K209			K162	K162
			K214			K166	K166
						K214	K209
							K214

Using free protein, a higher number of lysines was modified for almost each case. Consequently, solid-phase biotinylation reduces the extent of biotinylation which is probably due to steric hindrance and reduced accessibility of reaction sites. In addition, temporary fixation of the substrate onto the solid-phase strongly reduces its mobility and, therefore, the collision probability between the protein and NHS-PEG. In consequence the reaction rate is reduced which may also lead to a smaller number of modified lysines. For a preferably complete conversion high excesses of NHS-biotin are thus necessary. The difference in the number of modified lysines ranged from two to four. On the one hand, this indicates a lower efficiency for the solid-phase reaction. On the other hand, this can be regarded as a step toward a more specific biotinylation reaction *via* NHS-biotin, since a more homogenous product is formed. This can be advantageous for certain applications. However, as MALDI-TOF as qualitative method was chosen for analysis, no quantitative conclusions can be drawn which would deliver further information.

The solid-phase reaction also did not only reduce the number of modified lysines but also changed their composition: K101 and K166 were only modified in solution and not in the solid-phase reaction. Thus, by use of the solid-phase reaction certain lysines can be excluded from the modification reaction which can be useful for reaction control. Possible causes for this are shielding of amino acids by the particle surface and steric effects close to the carrier. Maiser et al. (2015) observed a change in the isoform composition after comparing solid-phase PEGylation with PEGylation of free protein. They were able to lead this difference back to steric effects. However, the PEG applied in this study was larger (kDa) than the biotin used here so that steric hindrance has a smaller effect.

To our knowledge, these experiments represent the first reaction cascade combining protein modification and enzymatic digest, thus sample preparation, in a compartmented microfluidic device.

#### Modification at Different pH-Values

In a second set of experiments the influence of the pH-value on solid-phase biotinylation was investigated. As the NHS-group is subject to pH-dependent hydrolysis, which might falsify the results, we first investigated a method for an *in situ* change of the pH in the reactor. By changing the pH-value right before the modification reactions, a pH-dependent hydrolysis of NHS can be minimized.

**Figure 4:**
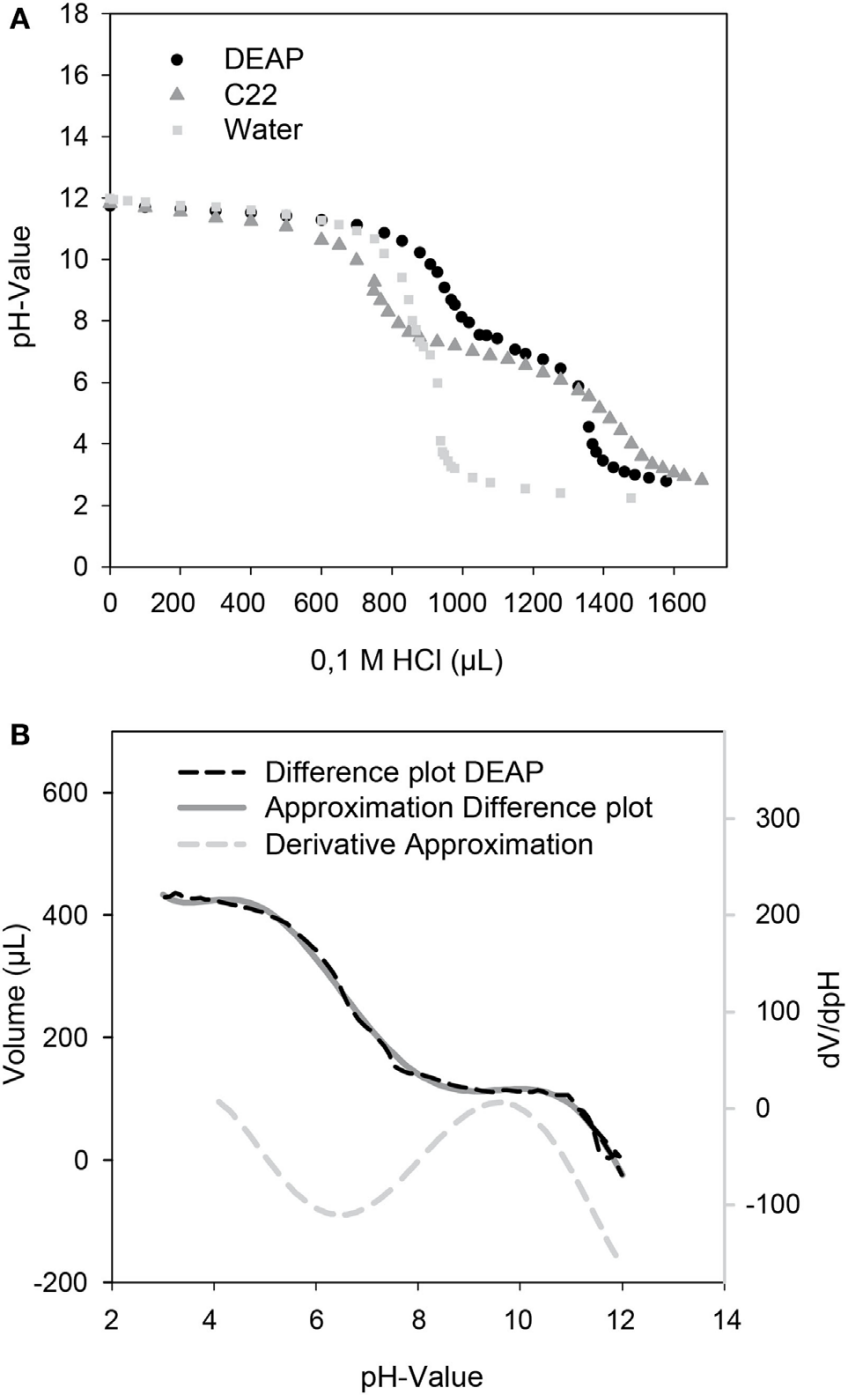
**(A)** Titration curves of the DEAP, C22 particles, and water as control. 20 mg particles in 10 mL of a solution of 0.01 M NaOH were titrated with 0.1 M HCl. **(B)** For determination of an additional pKa of the DEAP particles, a difference plot of the DEAP and water titration curve was created and derived.

A unique feature of the developed microfluidic reactor is its capability to transfer magnetic micro particles in and out of the compartmented plugs in which the reaction takes place. Therefore, besides serving as carriers for solid-phase protein modification, other types of magnetic particles can also be used for pH adjustment. For these magnetic micro particles with carboxy or DEAP, functionalities were used as source or sink of protons. The titration curves for both particles are given in Figure 4. Both particle types possess a plateau between pH 6 and pH 7.5 which leads to a pKa of ca. 6.75. This pKa results from the carboxy groups of the particles. This pKa can also be determined for the DEAP particles as they are synthesized on the basis of the carboxy particles (Müller et al., 2011). It is likely that the conversion was not complete, so that carboxy groups are still present on the surface. Starting at around pH 10, the titration curves of the particle suspensions separate from the titration curve for water. For the DEAP particles this indicates buffering by amine-groups. For the DEAP particles an additional pKa of 9.3 was determined by use of the method by Frimmel et al. (1985). From the titration curves, it can be seen that because of their functional groups the particles are suitable for alteration of the pH-value inside the reactor. Depending on the amount of particles applied, particles with carboxy groups in the protonated form are able to decrease the pH down to about pH 6. In contrast, particles with DEAP groups can increase the pH up to approx. pH 10. The limited pH-range is caused by the use of weak acids and bases as functional groups. If functional groups with strong dissociation behavior had been used instead, the accessible pH-range could be strongly enhanced.

**Figure 5:**
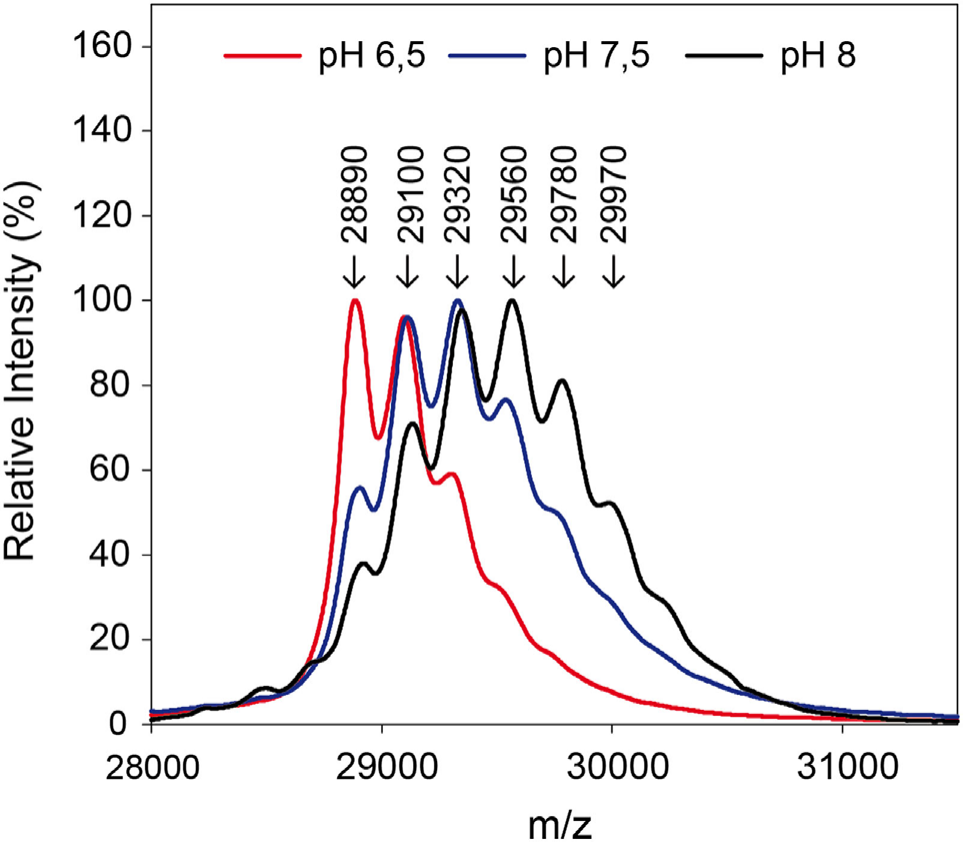
Mass spectra after biotinylation of eGFP at different pH-values. A 20-fold excess of biotin compared to the used 0.3 mg protein was applied.

By use of the *in situ* pH-control, solid-phase biotinylation was performed at the pH-values 6.5, 7.5, and 8.0 in the reactor with a 20-fold biotin-excess as described in Section “Protein Modification Reactions.” After 5 min reaction time, the reaction plug was withdrawn from the reactor into the fractionation area. Half of the particles were separated and protein was eluted for MALDI measurements in linear mode. The results of the measurements are shown in Figure 5. Subsequently, the enzymatic digest was performed with the remaining particles.

The measurements in linear mode allow for a first characterization of the reaction: a lower pH-value leads to a reduced modification. Fewer biotins were bound to the protein than for higher pH-values, as becomes clear from the lower molecular weights. In the solid-phase reaction, a mass of up to 29,970 Da was measured which corresponds to five coupled biotin molecules.

The ratio of modified to unmodified protein (mass of about 28,890 Da) gives a relative impression of the extent of the yield. The higher the pH-value the less unmodified protein is present. Apparently, the reaction is accelerated at higher pH-values. This is in accordance with literature (Nojima et al., 2009; Maiser et al., 2014). For more detailed information, the samples were also digested by pepsin and then measured by MALDI in reflector mode and with MS/MS, in order to identify the modified amino acids. The modified lysines are given in Table 2. As in the experiment with different biotin-excesses, the modified biotins appear in a sorted way: If a lysine is modified at a lower pH-value, it also is for a higher value. It should be mentioned that this is not necessarily the case, e.g., for coupling with aldehydes, it has been observed that the modified lysines alter depending on the pH-value (Maiser et al., 2014).

**Table 2:** Modified lysines by use of different pH-values for immobilized and free eGFP.

**Different pH-values**			**Different pH-values**		
**Solid-phase reaction**			**Reaction with free protein**		
pH 6.5	pH 7.5	pH 8.0	pH 6.5	pH 7.5	pH 8.0

K101	K101	K26	K26	K26	K26
K107	K107	K85	K107	K85	K85
K113	K113	K101	K113	K107	K101
K140	K126	K107	K126	K113	K107
K209	K131	K113	K140	K126	K113
K214	K140	K126	K209	K140	K126
	K156	K131	K214	K156	K131
	K158	K140		K158	K140
	K162	K156		K162	K156
	K214	K158		K209	K158
		K162		K214	K162
		K209			K209
		K214			K214

As can be seen in Table 2, the modified lysines also indicate that a higher pH-value leads to an increased biotinylation both for free and immobilized protein. Regarding the modified lysines, the differences between the solid-phase reaction and reaction with dissolved protein are less significant than for the experiments using different excesses of NHS-biotin. This might be due to the shorter reaction time used for the pH-experiments (5 min instead of 60 min for different excesses). A longer reaction time might lead to a higher number and a higher variety of modified amino acids.

The number of coupled biotins is not equal for the measurement in linear and in reflector mode. One reason for this is the formation of isomers: Despite the same number of bound biotins, different eGFP molecules apparently have the biotins bound to different positions. As consequence, more modified lysines were measured altogether than bound per eGFP in the reflector mode.

## Conclusion

In this study, we were able to enhance and apply a compartmented, microfluidic reactor for protein modification reactions and subsequent sample preparation for detailed analysis of the reaction products. On the one hand, it was possible to parallelize the reactor so that multiple experiments were feasible in parallel. On the other hand, the reactor could be applied for the screening of different reaction conditions: pH-value, and the excess of NHS-biotin was varied. Modification was directly coupled with enzymatic digest and, thus, with sample preparation. A detailed analysis of the modified lysines was possible by use of mass spectrometric methods.

As expected, a higher excess of NHS-biotin and a higher pH-value lead to an increased conversion. Additionally, the modified lysines appeared to react in a specific order. Furthermore, it was observed that the modification of immobilized proteins varied from the respective modification of free proteins in the amount of modified lysines, but partly also in the location of the modification. Both aspects might be useful for process control.

Reactant concentration and pH-value were screened in this study, but also other conditions like reaction duration are possible. The same is valid for the reaction types which can be studied: Various modification reactions in aqueous buffer are thinkable in the reactor and besides biotinylation also PEGylation has been investigated in a former study with a simpler version of the reactor. As was additionally shown, enzymatic solid-phase reactions are also possible within the system. Thus, in future a wide range of more complex solid-phase reactions can be performed in the reactor, like, e.g., glycosylation of immobilized proteins.

## Author Contributions

MF, the leader of the research group, contributed to the work by giving the idea for this project, helping in the case of scientific problems and writing parts of the manuscript. RF was the scientist responsible for planning and performing experiments and also writing the main part of the manuscript. JD performed a great part of the experiments and gave valuable input during writing the manuscript.

## Conflict of Interest Statement

The authors declare that the research was conducted in the absence of any commercial or financial relationships that could be construed as a potential conflict of interest. The reviewer, MJ, and handling editor declared their shared affiliation.
