# Supplementary material for: Automated Solid-Phase Protein Modification with Integrated Enzymatic Digest for Reaction Validation: Application of a Compartmented Microfluidic Reactor for Rapid Optimization and Analysis of Protein Biotinylation
Source: Front Bioeng Biotechnol. 2017 Nov 13;5:72. doi: 10.3389/fbioe.2017.00072 (PMC5693853; doi:10.3389/fbioe.2017.00072)
Supplement: Supplementary file 1 [file Data_Sheet_1.DOCX]

Automated solid-phase protein modification with integrated enzymatic digest for reaction validation: Application of a compartmented microfluidic reactor for rapid analysis of protein biotinylation

Regina Fraas^1^, Juliane Diehm^1^, Matthias Franzreb^1*^

^1^ Institute of Functional Interfaces, Karlsruhe Institute of Technology, Karlsruhe, Germany

*** Correspondence:**Corresponding Author
Matthias.franzreb@kit.edu

# Supplementary Data

eGFP sequence:

MTMITHHHHHHGSSKGEELFTGVVPILVELDGDVNGHKFSVSGEGEGDATYGKLTLKFICTTGKLPVPWPTLVTTLTYGVQCFSRYPDHMKQHDFFKSAMPEGYVQERTIFFKDDGNYKTRAEVKFEGDTLVNRIELKGIDFKEDGNILGHKLEYNYNSHNVYIMADKQKNGIKVNFKIRHNIEDGSVQLADHYQQNTPIGDGPVLLPDNHYLSTQSALSKDPNEKRDHMVLLEFVTAAGITLGMDELYK
